# The relationship between adherence, life satisfaction, and acceptance of illness among patients with mood disorders

**DOI:** 10.1192/j.eurpsy.2025.1367

**Published:** 2025-08-26

**Authors:** D. Schneider-Matyka, K. Rachubińska, E. Grochans, A. Jeżuchowska, A. Reginia, M. Panczyk, A. M. Cybulska

**Affiliations:** 1 Department of Nursing, Pomeranian Medical University in Szczecin, Szczecin; 2 Department of Clinical Nursing, Wroclaw Medical University, Wrocław; 3 Department of Psychiatry, Pomeranian Medical University in Szczecin, Szczecin; 4 Department of Education and Research in Health Sciences, Medical University of Warsaw, Warszawa, Poland

## Abstract

**Introduction:**

Mood disorders are among the most prevalent and debilitating mental conditions in worldwide populations. Non-adherence to treatment recommendations may have serious consequences for patients with mood disorders. There are several methods to show whether the patient cooperates with the doctor and follows his recommendations, or whether he or she skips the prescribed doses of medications. These include objective methods, such as detecting the drug in the blood, urine or saliva by analysis or special markers, pill counting, electronic monitoring, and an electronic record of filled prescriptions. Subjective methods involve assessment of the patient, medical staff, and those in the patient’s immediate environment. The most commonly used subjective methods are an interview, filling out a questionnaire, and assessment by health care professionals and the patient’s relatives The problem arises when the patient inadvertently skips medications, which makes it difficult to assess adherence. This may be due to the severity of the disease and poorer cognitive function, and sometimes a change in daily routine.

**Objectives:**

The aim of the study was to identify factors influencing life satisfaction, disease acceptance and therapeutic adherence among people with mood disorders.

**Methods:**

This survey-based study included 103 people with mood disorders. It was performed using the author questionnaire, and standardized research tools, namely: the Adherence to Refills and Medication Scale (ARMS), the Acceptance of Illness Scale (AIS), the Beck Depression Inventory (BDI), and the Satisfaction with Life Scale (SWLS).

**Results:**

The level of life satisfaction decreased with an increase in the severity of depressive symptoms (βstd. = -0.665, p < 0.001). Mood disorder patients with more severe depressive symptoms had significantly higher scores on the adherence scale (βstd. = 0.290, p = 0.003). Patients with higher levels of depressive symptoms showed a lower level of acceptance of the disease (βstd. = -0.215, p < 0.001).

**Conclusions:**

1. The dosage of medications taken, and the severity of depressive symptoms determine life satisfaction of people with mood disorders.
2. Respondents with greater severity of depressive symptoms scored higher on the adherence scale, which means that they were more likely to be non-adherent to treatment recommendations. The type of mood disorder may affect patient adherence. Subjects with bipolar disorder showed higher and those with anxiety-depressive disorder—lower adherence than patients with depression.
3. Subjects with more severe depressive symptoms showed a lower degree of acceptance of the disease.

**Disclosure of Interest:**

None Declared

